# Transmission dynamics of Nipah virus in Bangladesh and India, 2001–2026: Systematic review and inference on reproduction number, offspring dispersion, and serial interval

**DOI:** 10.1371/journal.pntd.0014876

**Published:** 2026-11-10

**Authors:** Sol Kim, Vijayalaxmi V. Mogasale, Juan F. Vesga, Hyolim Kang, Laura Skrip, Sung-mok Jung, Ausraful Islam, Akira Endo, W. John Edmunds, Kaja Abbas

**Affiliations:** 1 Department of Infectious Disease Epidemiology and Dynamics, London School of Hygiene & Tropical Medicine, London, United Kingdom; 2 School of Tropical Medicine and Global Health, Nagasaki University, Nagasaki, Japan; 3 Department of Infectious Disease Epidemiology and Dynamics, Institute of Tropical Medicine, Nagasaki University, Nagasaki, Japan; 4 Modelling & Economics Unit, UK Health Security Agency, London, United Kingdom; 5 Saw Swee Hock School of Public Health, National University of Singapore, Singapore, Singapore; 6 Department of Preventive Medicine, Seoul National University College of Medicine, Seoul, Republic of Korea; 7 Infectious Diseases Division, icddr, b, Dhaka, Bangladesh; 8 Public Health Foundation of India, New Delhi, India; 9 National Institute of Infectious Diseases, Japan Institute for Health Security, Tokyo, Japan; The Pennsylvania State University, UNITED STATES OF AMERICA

## Abstract

**Background:**

Nipah virus (NiV) is a priority zoonotic pathogen causing high-fatality outbreaks. Early NiV outbreaks in Malaysia and Singapore had limited transmission beyond spillover events. However, since 2001, NiV outbreaks with person-to-person transmission have occurred in Bangladesh and India, driven by the NiV-Bangladesh genotype and NiV-India genotype. Our study aims to estimate the reproduction number, offspring dispersion, and serial interval governing NiV transmission in Bangladesh and India during 2001–2026.

**Methods:**

We conducted a systematic review of NiV outbreak investigations in Bangladesh and India, searching PubMed, Embase, Web of Science, and grey literature through 28 February 2026. Case-level offspring counts from 27 sources (323 cases across 67 outbreaks) were used as input to a hierarchical Bayesian negative binomial offspring distribution model. The serial interval was estimated by parametric distribution fitting to 137 transmission pairs. We conducted country-specific analysis and performed sensitivity analyses to evaluate the robustness of estimates.

**Results:**

Pooling across 67 outbreaks, we estimated a median reproduction number of 0.43 (95% CrI: 0.26–0.75), an offspring dispersion parameter of 0.04 (0.03–0.06), and a serial interval of 13.3 days (95% CI: 12.8–13.8). Country-specific median reproduction numbers were 0.47 (0.22–0.98) for India and 0.35 (0.19–0.63) for Bangladesh, and dispersion parameters were 0.03 (0.02–0.06) and 0.05 (0.03–0.09), respectively, indicating marked overdispersion in both settings.

**Conclusion:**

NiV transmission is self-limiting on average and highly overdispersed, suggesting that a disproportionate share of onward transmission arises from a small number of cases. This epidemiological profile supports targeted containment measures, including contact tracing and quarantine, for effective NiV outbreak control.

## Author summary

Nipah virus is a lethal infection that spreads from bats to people and, in some cases, from one person to another. Despite its severity as a high-consequence pathogen, there is no licensed vaccine or specific treatment. Since 2001, outbreaks have occurred almost every year in Bangladesh and India, which are the only places with person-to-person transmission. We gathered case reports from these outbreaks over twenty-five years and used a statistical model to measure how the virus spread between people. We found that, on average, each infected person passes the virus to fewer than one other person, so outbreaks tend to die out on their own without repeated introductions from animals. However, spread is heterogeneous: most people infect no one, while a small number infect many, occasionally producing large clusters. We estimated the typical time between one person falling ill and the next (serial interval) to be thirteen days. Together, these findings provide a better understanding of Nipah virus transmission and updated numbers needed to plan future prevention and control strategies for Nipah outbreaks.

## Introduction

Nipah virus (*Henipavirus nipahense*, NiV) is a zoonotic, negative-sense single-stranded RNA virus in the family *Paramyxoviridae*. It was first identified during the 1998–1999 Malaysia and Singapore outbreak, where transmission was primarily from infected pigs to pig farmers and abattoir workers [1,2]. Since 2001, recurrent outbreaks in South Asia have been characterised by repeated zoonotic spillover from *Pteropus* fruit bats, followed by person-to-person transmission [3,4]. In Bangladesh, the principal spillover route is consumption of raw or fermented date palm sap (‘tari’) contaminated by bat excretion, with additional contributions from contact with infected domestic animals such as pigs and cows [5–7]. Three genotypes circulate: NiV-Malaysia (NiV-M), NiV-Bangladesh (NiV-B), and NiV-India (NiV-I) [8–10]. NiV-I, closely related to NiV-B, has been associated with the recurring outbreaks in Kerala, India, since 2018 [8,10,11]. Compared with NiV-M, infections caused by NiV-B and NiV-I are associated with higher case-fatality risks and more frequent respiratory manifestations [7,12,13]. As of May 2024, at least 754 confirmed human cases had been documented across five countries in South and Southeast Asia, with overall case-fatality risks exceeding 70% in both Bangladesh and India, substantially higher than in the initial Malaysia and Singapore outbreaks [14,15].

NiV has been designated by the World Health Organization (WHO) and the Coalition for Epidemic Preparedness Innovations (CEPI) as a priority pathogen for research and development due to its epidemic potential [16–18]. While no dedicated therapeutic or licensed vaccine is currently available for NiV, several vaccine candidates have shown promising Phase-1 immunogenicity data. Of note, ChAdOx1 NipahB is entering Phase-2 trials with up to 100,000 investigational doses planned for rapid outbreak deployment [19,20]. Better epidemiological understanding of NiV will be critical for overall preparedness and for clinical trial planning for novel vaccine and therapeutic candidates.

Unlike the 1998–1999 Malaysia and Singapore outbreaks, which involved no person-to-person transmission and have not recurred, NiV outbreaks in Bangladesh and India have occurred almost every year since 2001 and are characterised by person-to-person transmission events. Bangladesh and India therefore represent the only settings in which the person-to-person transmission dynamics of NiV can be empirically studied, making them the focus for estimating epidemiological parameters relevant to pandemic risk assessment and vaccine deployment planning. Key parameters of interest characterise the offspring distribution, defined as the distribution of the number of secondary cases generated by a single infected individual. This is typically fit by applying a negative binomial distribution to available offspring distribution data [21].

The negative binomial offspring distribution is governed by the reproduction number (*R*) and the dispersion parameter (*k*). *R* is the mean of the offspring distribution and quantifies average transmissibility. It defines the epidemic threshold: transmission is self-sustaining when *R* > 1 and subcritical, tending to extinction, when *R* < 1. The dispersion parameter (*k*) governs how unevenly transmission is distributed across cases—when *k* is large, most cases transmit similarly to the average; when *k* is small, the majority of cases generate no or very few secondary infections while a minority are responsible for most onward transmission. Together with the serial interval, the interval between symptom onset in an infector and the person they infect (infectee), these parameters characterise the transmission dynamics that inform decisions on the prevention and control of Nipah outbreaks and plan for implementation strategies for new NiV vaccines in the development pipeline.

To date, published estimates of both parameters derive almost exclusively from Bangladesh and are lacking for India [6,15,22–24]. To address this evidence gap, we systematically reviewed publicly available literature on NiV outbreaks in Bangladesh and India, and estimated the reproduction number, offspring dispersion, and serial interval of NiV transmission during 2001–2026.

## Methods

### Ethics

This study used data from published outbreak investigations and publicly available reports. Ethical approval and informed consent were not required.

### Search strategy and study selection

We searched PubMed, Embase, and Web of Science through February 28, 2026, using terms related to Nipah virus and epidemiology, with no date or language restrictions (Table B in S1 Appendix). WHO Disease Outbreak News and the icddr,b Health and Science Bulletin were also searched as additional sources [25,26]. The review is registered with PROSPERO (CRD42025641668).

We included studies that reported observational human NiV outbreaks. We excluded studies restricted to animals, systematic reviews and other review articles, and modelling studies (Table C in S1 Appendix). Title and abstract screening was performed by one reviewer (SK); full-text review was conducted independently by two reviewers (SK, VVM), with disagreements resolved by discussion and consensus.

### Data extraction

Data were extracted by one reviewer (SK) and independently verified by a second reviewer (VVM), using a standardised data extraction spreadsheet, on study year, setting, infector-infectee pairs, dates of symptom onset per case, and other variables (Table D in S1 Appendix). When multiple studies reported the same outbreak, we prioritised the source with the more complete case ascertainment and detailed infector-infectee data (Text A in S1 Appendix). For this analysis, an outbreak was defined as one or more confirmed or probable NiV case(s)—classified according to the case definitions applied in the original source study—that were geographically and temporally clustered.

### Quality assessment

Risk of bias was assessed by two independent reviewers (SK, VVM) using JBI critical appraisal tools for case-control studies, case series, and case reports (Table F in S1 Appendix) [27]. Discrepancies were resolved by discussion.

### Transmission pair reconstruction

Transmission pairs were reconstructed from the included outbreak investigation reports at the case level, recording infector–infectee links, symptom onset dates, and secondary case counts. Each human case was assigned an offspring count equal to the number of person-to-person transmission events in which they subsequently appeared as the infector. Cases generating no further transmission were assigned a null offspring count. Non-human or environmental sources (e.g., bats, date palm sap) appearing only as primary infectors were not used to construct the offspring distribution (Text B and C in S1 Appendix).

### Offspring distribution model

To better account for variation in transmission context across settings, time periods, and countries, we fitted a hierarchical negative binomial model in which each outbreak *j* has its own reproduction number (*R*_*j*_) drawn from a lognormal distribution, while a single dispersion parameter *k* is shared across all cases and outbreaks. This structure allows the reproduction number to vary across outbreaks while partially pooling information across them, and separates two distinct sources of variation in the data: individual-case heterogeneity in transmission (*k*) and between-outbreak heterogeneity in reproduction number (*σ*_*R*_, the log-scale standard deviation of the lognormal distribution). The model is specified as follows:

For case *i* in outbreak *j*, the number of secondary cases *X*_*ij*_ follows:


$$\begin{array}{c} X_{ij}|R_{j},k\sim NegBin(R_{j},k) \end{array}$$

where *i* = 1,...,*n*_*j*_ index cases for *n*_*j*_ cumulative cases in outbreak *j*. On the log scale:


$$\begin{array}{c} log(R_{j})\;\sim Normal(\mu_{R},\sigma_{R}^{2}) \end{array}$$

where *μ*_*R*_ is the mean of log(*R*_*j*_) across outbreaks. From the fitted lognormal distribution of outbreak-specific reproduction numbers, two summary measures were derived: the median, *R* (median) = exp(*μ*_*R*_), representing the reproduction number of a typical outbreak and used as the primary estimate throughout; and the arithmetic mean, *R* (mean) = exp(*μ*_*R*_ + *σ*_*R*_^2^/2), the average reproduction number across outbreaks, reported for downstream modelling applications. Both quantities were computed for each posterior draw and summarised as posterior medians with 95% credible intervals.

Three prior specifications (P1: weakly informative reference; P2: epidemiologically informed, permissive between-outbreak heterogeneity; and P3: epidemiologically informed, conservative between-outbreak heterogeneity) were evaluated. Parameters were estimated in a Bayesian framework using the No-U-Turn Sampler (NUTS) in Stan via the *cmdstanr* package in R (version 4.5.2), using a non-centred parameterisation [28–30]. Full prior specifications and sampler settings are provided in Text D in S1 Appendix, and posterior predictive check definitions in Text F in S1 Appendix.

Country-specific analyses fitted the same hierarchical negative binomial model independently to Bangladesh and India subsets under P2 (epidemiologically informed, permissive between-outbreak heterogeneity) prior specification. Between-country differences were assessed by contrasting the independent country posteriors on the log scale, summarised as the posterior probability of difference and the median India-to-Bangladesh ratio (95% credible interval). Sensitivity analyses examined: (1) restriction to outbreaks with person-to-person transmission (SA-O-P2P); (2) exclusion of the two manually inferred index cases (SA-O-MI) (Text C in S1 Appendix); (3) Bangladesh surveillance-era stratification by pre- and post-2007 periods (2001–2006 vs. 2007 onwards), corresponding to the introduction of hospital-based sentinel surveillance (SA-O-B1, SA-O-B2); (4) For Bangladesh, we set 137 cases to represent uncaptured single-case spillovers, derived from the difference between the 211 cases in the current dataset and the 348 cases reported by the Institute of Epidemiology, Disease Control and Research (IEDCR) (SA-O-B3) [31]; and (5) we reparameterised the variance-to-mean ratio to hold constant across outbreaks, with *ψ* = *R*/*k* shared in place of *k* (SA-O-CV). Methodological details of the sensitivity analyses are provided in the Appendix (Text E in S1 Appendix).

### Serial interval estimation

The serial interval was defined as the number of days between symptom onset in the infector and symptom onset in the infectee. Pairs were included if both onset dates were available; no negative serial intervals were present in the data. Gamma, lognormal, and Weibull distributions were fitted by maximum likelihood using the *fitdistrplus* package in R, with distribution selection based on Akaike Information Criteria (AIC) [32,33]. Uncertainty in the fitted mean and standard deviation was quantified by non-parametric bootstrap with 1,000 resamples, refitting the selected distribution within each replicate. Country-specific fitting was conducted independently within each subset with independent distribution selection. Two sensitivity analyses (referred to as SA-S1 and SA-S2) were conducted. The first restricted the analysis to confirmed transmission pairs by excluding infectees with multiple candidate infectors (SA-S1). The second involved a separate analysis of Bangladesh transmission pairs from pre- and post-2007 periods to assess the sensitivity of the estimate to surveillance changes (SA-S2).

## Results

### Study selection and quality assessment

A total of 2,686 unique articles were identified through the review of published literature, and 26 additional data sources were identified through repositories, grey literature sources, and review of reference lists (Fig 1). 34 references met the inclusion criteria after full-text review [25,26,34]. This included one WHO SEARO Nipah Webinar report (2026) which served as a supporting data source for the 2025–2026 West Bengal outbreak [35]. During data extraction, seven studies were excluded because they reported the same outbreaks with less complete case ascertainment than the selected primary sources (Text A in S1 Appendix). Ultimately, 27 sources were used for data extraction (Table E in S1 Appendix).

**Fig 1:**
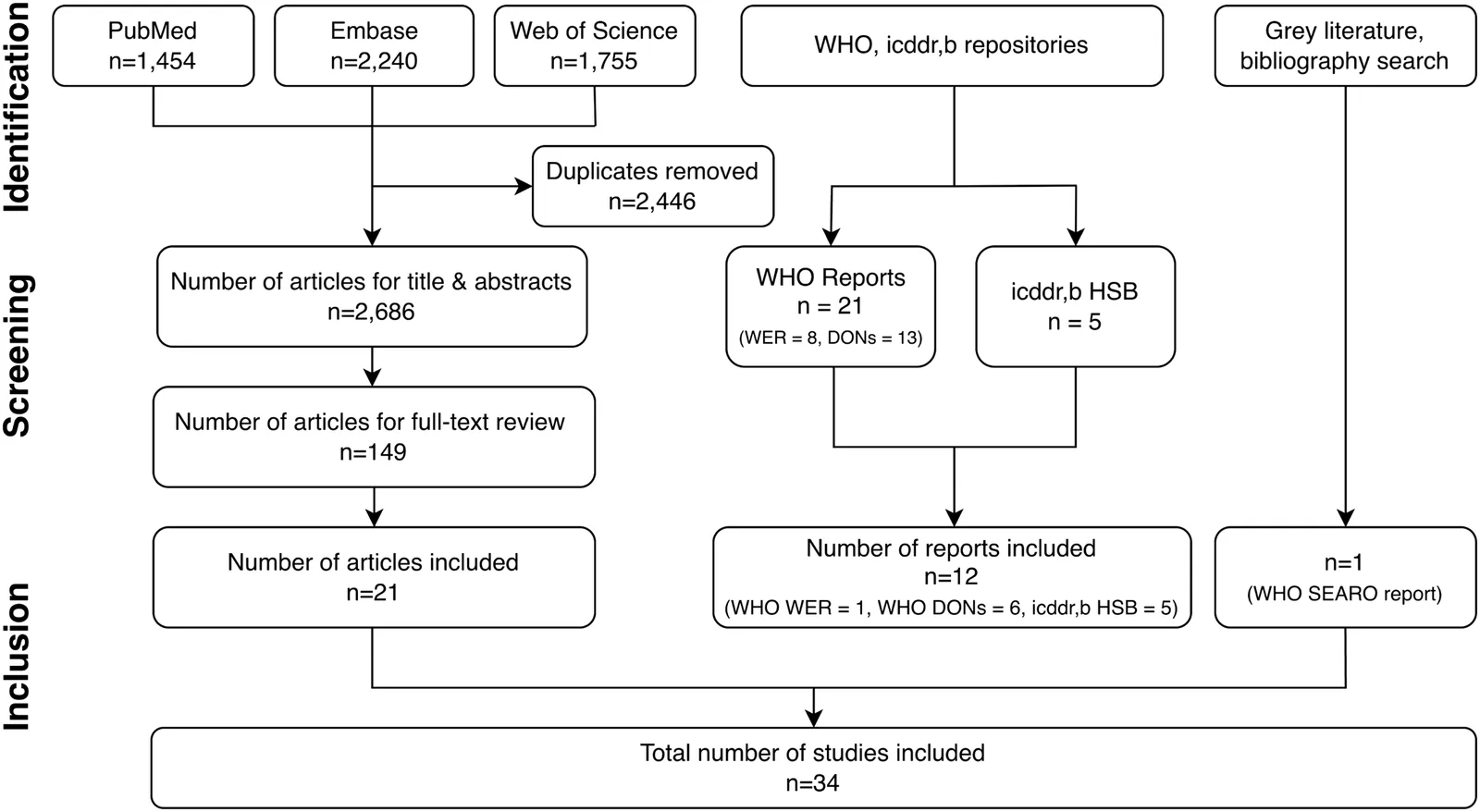
Systematic review of Nipah virus outbreaks in Bangladesh and India. PRISMA (Preferred Reporting Items for Systematic reviews and Meta-Analyses) flow diagram showing the number of records at each stage of identification, screening, and inclusion.

The included sources comprised nine case-control studies [5,36–43], 10 case series [11,44–52], and two case reports [53,54]. Across study types, included references were generally considered of moderate or high quality, with one exception [11]. The most consistently underreported items across study types were consecutive and complete inclusion of participants and identification and management of confounding (Table F in S1 Appendix).

### Outbreak summary

The 34 articles documented 67 discrete outbreaks spanning 2001–2026 (Table 1): 54 in Bangladesh (211 cases including 166 deaths) and 13 in India (112 cases including 80 deaths) (Fig 2). Outbreaks were classified into three types: (1) clusters with at least one documented person-to-person transmission pair (n = 21 outbreaks with 245 cases); (2) single-case spillover events with no onward transmission (n = 40 outbreaks); and (3) multi-case spillover clusters with no person-to-person transmission involved, in which cases occurred in the same area within a defined period, suggesting a possible shared zoonotic source such as a common contaminated date palm sap (n = 6 outbreaks with 38 cases) (Fig A in S1 Appendix). Transmission chain depth, defined as the number of person-to-person transmission events following the index case (Generation 0), was characterised for the 21 outbreaks with documented person-to-person transmission (16 outbreaks, 141 cases, in Bangladesh; five outbreaks, 104 cases, in India) (Table G in S1 Appendix). Most outbreaks (17/21, 81%) were limited to a single transmission generation beyond the index case (depth = 1); four (19%) extended further, with a maximum depth of four (five generations; 2004 Faridpur). In Bangladesh, 14 of 16 outbreaks (87.5%) reached depth 1, and the remaining two outbreaks reached depths of two (2001 Meherpur) and four (2004 Faridpur). In India, three of five outbreaks (60%) reached depth 1, and the remaining two outbreaks reached a depth of two (2001 Siliguri and 2018 Kerala).

**Table 1:** Nipah virus outbreaks in India and Bangladesh.

Year	Region	Start date	End date	Cases^*^	Confirmed^†^	Deaths^‡^	Sources
**India**							
2001	Siliguri (West Bengal)	Jan 31	Feb 23	67^§^	11	45	Chadha et al. (2006), Harit et al. (2006) [44,47]
2007	Nadia (West Bengal)	Apr 9	Apr 23	5	3	5	Arankalle et al. (2011) [11]
2018	Kozhikode (Kerala)	May 2	May 29	23	18	21	Arunkumar et al. (2019), Pallivalappil et al. (2020) [45,50]
2019	Ernakulam (Kerala)	Early June	Early June	1	1	0	Ramachandran et al. (2022) [51]
2021	Kozhikode (Kerala)	Aug 29	Aug 29	1	1	1	Yadav et al. (2022) [53]
2023	Kozhikode (Kerala)	Sep 12	Sep 15	6	6	2	As et al. (2024), WHO DONs^¶^ (2023) [46,55]
2024	Malappuram (Kerala)	July 10	July 10	1	1	1	Sahay et al. (2025) [52]
2024	Malappuram (Kerala)	Sep 5	Sep 5	1	1	1	Sahay et al. (2025) [52]
2025	Malappuram (Kerala)	Apr 25	Apr 25	1	1	0	WHO DONs^¶^ (2025) [56]
2025	Malappuram (Kerala)	Jun 23	Jun 23	1	1	1	WHO DONs^¶^ (2025) [56]
2025	Palakkad (Kerala)	Jun 25	Jun 25	1	1	0	WHO DONs^¶^ (2025) [56]
2025	Palakkad (Kerala)	Jul 6	Jul 6	1	1	1	WHO DONs^¶^ (2025) [56]
2025	North 24 Parganas (West Bengal)	Mid-Dec 2025	Late Dec 2025	3	2	2	WHO DONs^¶^ (2026), WHO SEARO report (2026) [35,57]
**Bangladesh**							
2001	Meherpur	Apr 20	May 20	13	4	9	Hsu et al. (2004), icddr,b HSB^**^ (2003) [5,26]
2003	Naogaon	Jan 11	Jan 28	12	4	8	Hsu et al. (2004), icddr,b HSB^**^ (2003) [5,26]
2004	Rajbari	Jan 11	Jan 16	12	4	10	icddr,b HSB^**^ (2004), Montgomery et al. (2008), WHO WER^††^ (2004) [26,38,58]
2004	Faridpur	Feb 19	Apr 17	36	23	27	Gurley et al. (2007), WHO WER^††^ (2004) [36,58]
2004–2005	Tangail	Dec 31, 2004	Jan 16, 2005	12	3	11	icddr,b HSB^**^ (2005), Luby et al. (2006) [26,37]
2007	Thakurgaon	Jan 21	Feb 8	7	5	3	Homaira et al. (2010)a [39]
2007	Kushtia	Mar 17	Apr 2	8	3	5	Homaira et al. (2010)b [40]
2008	Manikganj	Feb 20	Feb 23	4	2	4	Rahman et al. (2012) [41]
2008	Rajbari	Feb 20	Feb 25	6	2	5	Rahman et al. (2012) [41]
2010	Faridpur^‡‡^	Jan 7	Jan 25	17^§§^	13	14	Sazzad et al. (2013) [42]
2011	Lalmonirhat	Jan	Feb	22	10	21	icddr,b HSB^**^ (2011), Chakraborty et al. (2016) [26,43]
2011	Rangpur	Jan 28	Mar 1	8	4	5	icddr,b HSB^**^ (2012), Chakraborty et al. (2016), Islam et al. (2016) [26,43,49]
2011	Dinajpur	NA	NA	5	2	4	Chakraborty et al. (2016) [43]
2011	Rajbari	NA	NA	2	1	2	Chakraborty et al. (2016) [43]
2011	Nipah-belt districts and Comilla^¶¶^	Jan	Mar	6	6	6	Chakraborty et al. (2016) [43]
2012	Rajshahi	Feb	Mar	3	2	2	Islam et al. (2016) [49]
2014	Rangpur	Jan	Feb	4	3	2	Islam et al. (2016) [49]
2013–2014	Faridpur, Rajshahi, Rangpur^***^	Dec 2013	Apr 2014	16	12	13	Hassan et al. (2018), Islam et al. (2016) [48,49]
2023	Rajbari^†††^	Jan 20	Jan 25	4	4	3	Rahman et al. (2025), WHO DONs^¶^ (2023) [54,59]
2023	Narsingdi, Shariatpur, Naogaon, Natore, Pabna, Rajshahi^‡‡‡^	Jan 4	Feb 13	7	7	5	WHO DONs^¶^ (2023) [59]
2024	Manikganj	Jan 11	Jan 11	1	1	1	WHO DONs^¶^ (2024) [60]
2024	Shariatpur	Jan 28	Jan 28	1	1	1	WHO DONs^¶^ (2024) [60]
2025	Pabna	Jan 25	Jan 25	1	1	1	WHO DONs^¶^ (2025) [61]
2025	Bhola	Feb 13	Feb 13	1	1	1	WHO DONs^¶^ (2025) [61]
2025	Faridpur	Feb 17	Feb 17	1	1	1	WHO DONs^¶^ (2025) [61]
2025	Naogaon	Aug 3	Aug 3	1	1	1	WHO DONs^¶^ (2025) [61]
2026	Naogaon	Jan 21	Jan 21	1	1	1	WHO DONs^¶^ (2026) [62]

* Cases: total number of confirmed, probable, and suspected cases, including those who died. ^†^ Confirmed: laboratory-confirmed cases (subset of Cases). ^‡^ Deaths: all deaths regardless of diagnostic classification; most deaths were probable or suspected cases, with a minority laboratory-confirmed. ^§^ Case count for the Siliguri 2001 outbreak increased by 1 (66 from Harit *et al*. (2006); 67 in the dataset). The primary case, though unidentified, has a precisely attributed offspring count of 9 from the transmission diagram and text description, but was not counted as a case in the source study. Excluding this case would discard a valid observation of an unusually high offspring count, biasing the offspring distribution toward underdispersion. We therefore include this case as an infector with an offspring count of 9, with an unknown onset date, and an unknown transmission setting (coded accordingly). ^¶^ World Health Organization Disease Outbreak News. ** icddr,b Health and Science Bulletin. ^††^ World Health Organization Weekly Epidemiological Record. ^‡‡^ Includes two clustered outbreaks and six sporadic outbreaks. ^§§^ Case count for Faridpur 2010 increased by 1 (16 from Sazzad *et al*. (2013); 17 in the dataset). The article suggested the possibility of a common infector for the two confirmed cases (one admitted patient with no history of date palm sap consumption and one physician). An unidentified NiV-infected case-patient in the same adult male ward, given the context, is considered highly plausible and included in the dataset as an unknown primary case. ^¶¶^ First NiV case in the eastern area of Bangladesh. Six sporadic outbreaks with spillover only. *** Includes three clustered outbreaks and four sporadic outbreaks. ^†††^ Includes one clustered outbreak and two sporadic outbreaks. ^‡‡‡^ Includes seven sporadic outbreaks.

**Fig 2:**
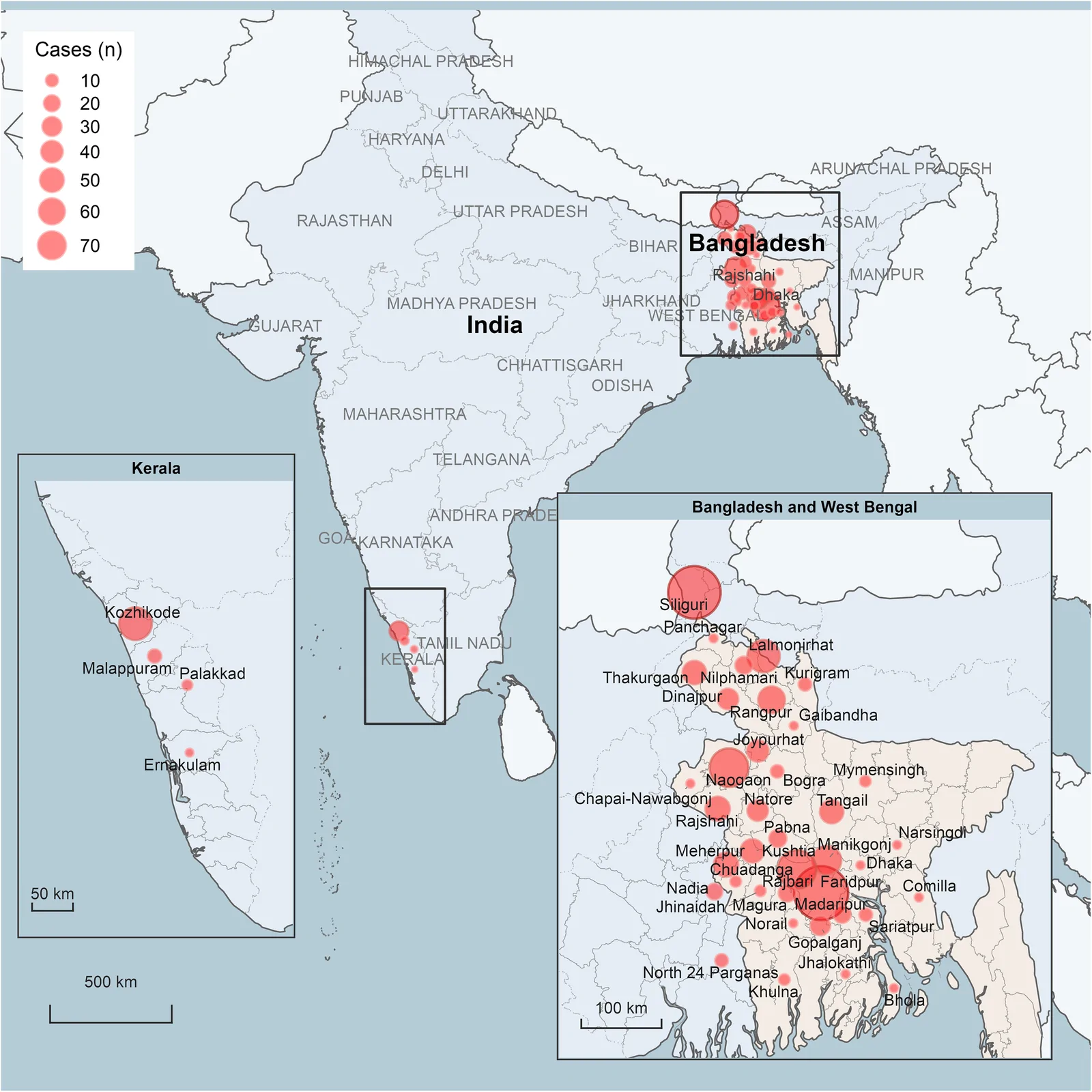
Geographic distribution of Nipah virus outbreaks in Bangladesh and India. Point size reflects the total number of cases per district from 2001 to 2026. Bangladesh totals during 2001–2026 are from the Institute of Epidemiology, Disease Control and Research (IEDCR) district-year counts (348 cases in 35 districts; https://iedcr.gov.bd/). India totals are retrieved by the present study (111 cases in 7 districts). Cases with unknown location are omitted from the map [n = 1, India (2018)]. Locations are district centroids. Natural Earth 1:10m Cultural Vectors (public domain; naturalearthdata.com). District boundaries are geoBoundaries gbOpen ADM2, CC BY 4.0 (geoboundaries.org).

We also examined the setting in which person-to-person infection occurred. Of the 245 cases in these 21 person-to-person outbreaks, 157 (64%) were secondary cases (generation ≥ 1), and 88 (36%) were generation 0 index or spillover cases. Among the 157 person-to-person secondary cases, 49% were infected in hospital, 21% in households, 19% in an unknown setting, and 11% in the community. This pattern differed sharply by country: 93% of Indian person-to-person infectees were hospital-acquired (71/76), compared with 7% in Bangladesh (6/81). Most infections occurred at generation 1 (90/157). Hospital transmission contributed many cases at generations 1–2 but did not produce the longest chains. The only outbreak with depth 4 (2004 Faridpur) was community- and unknown-dominated. Household infections were almost always a single step from the index (94% at generation 1) and rarely led to further transmission.

For Bangladesh, the 211 cases ascertained for transmission pair reconstruction in this analysis represent 61% (211/348) of the 348 cases reported by surveillance data for the full 2001–2026 (February) period (Fig 3) [7,14]. Data on the remaining cases could not be ascertained since discrete outbreak investigation reports during 2015–2022 were not identified from publicly accessible publications [6,7]. While the number of cases reported through the Bangladesh surveillance system is publicly available, that source does not provide outbreak-level information required for further analysis.

**Fig 3:**
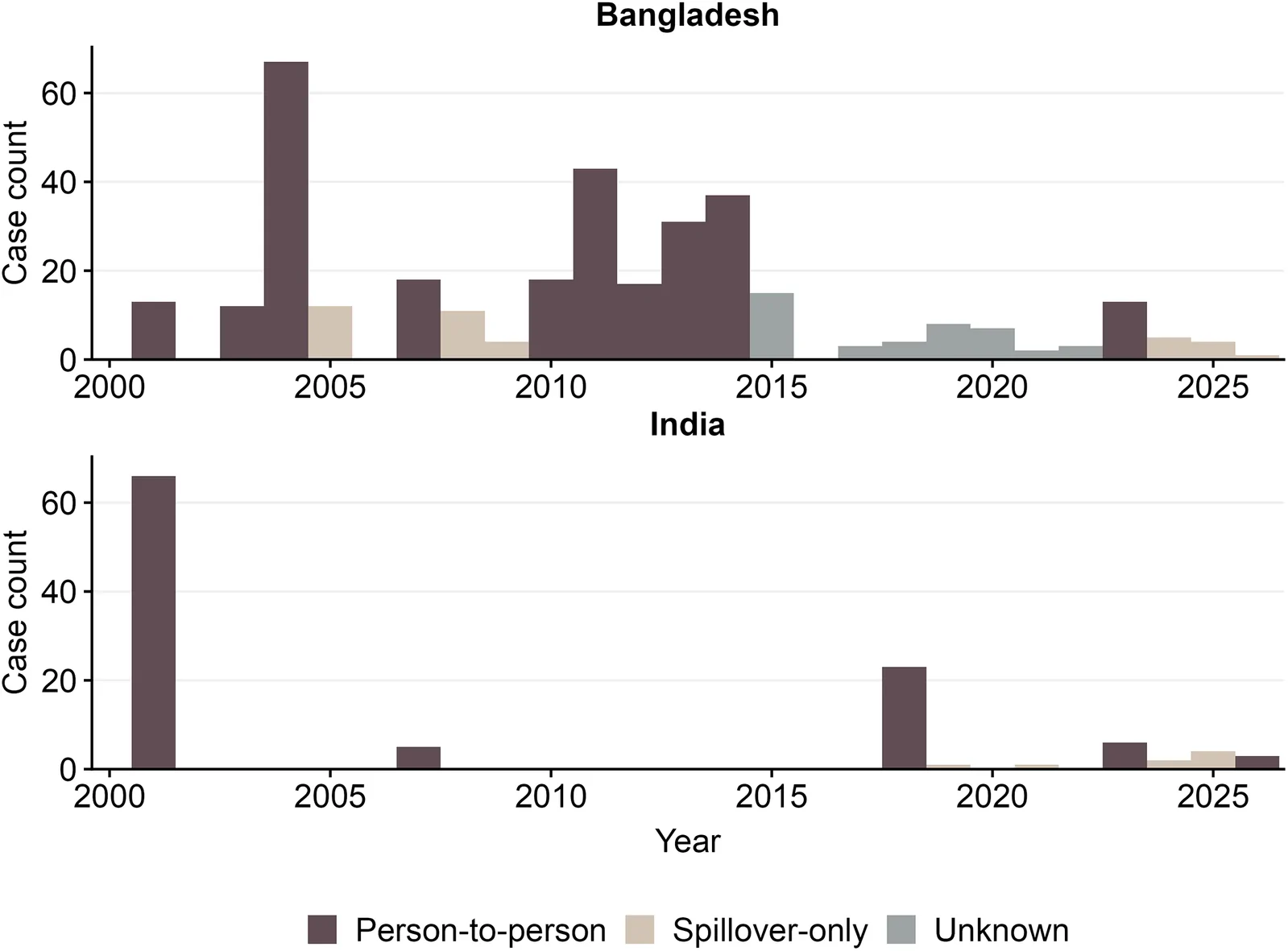
Nipah virus case counts, Bangladesh and India, 2001–2026. Confirmed and probable Nipah virus cases per year are shown. Colour indicates whether person-to-person transmission was documented in at least one outbreak in that year (dark taupe), all outbreaks were single-spillover cases (pale taupe), or transmission classification could not be determined from available sources (grey; 2015–2022, Bangladesh; data source: Institute of Epidemiology, Disease Control and Research (IEDCR), https://iedcr.gov.bd/).

### Reproduction number and offspring dispersion

Across 323 cases from 67 outbreaks, the hierarchical negative binomial model under prior set P2 (epidemiologically informed, permissive between-outbreak heterogeneity) yielded median *R* = 0.43 (95% CrI: 0.26–0.75) (Table 2). The mean *R*, reported for downstream modelling applications requiring an average over the outbreak distribution, was 0.47 (0.28–0.90). The offspring dispersion parameter *k* = 0.04 (0.03–0.06) indicates high individual-level heterogeneity in transmission (Table 2). Under priors P1–P3, estimates of median *R* and *k* were largely data-driven, whereas the between-outbreak heterogeneity parameter *σ*_*R*_ was weakly identified, with a credible interval that could not distinguish negligible from substantial between-outbreak heterogeneity (Table 2). Details on prior sensitivity and model validation results are provided in the Appendix (Text D and F; Table H; Figs B and E in S1 Appendix).

**Table 2:** Transmission parameter estimates for reproduction number, offspring dispersion parameter, and serial interval.

Stratum	Outbreaks	Cases	Reproduction number *R* (median) [95% CrI]	Reproduction number *R* (mean) [95% CrI]	Between-outbreak heterogeneity *σ*_*R*_ [95% CrI]	Offspring dispersion parameter *k* [95% CrI]	Serial Interval mean, days [95% CI] (n pairs)
Pooled	67	323	0.43 [0.26–0.75]	0.47 [0.28–0.90]	0.28 [0.01–1.00]	0.04 [0.03–0.06]	13.3 [12.8–13.8] (n = 137)
Bangladesh	54	211^*^	0.35 [0.19–0.63]	0.40 [0.23–0.87]	0.39 [0.02–1.24]	0.05 [0.03–0.09]	12.8 [12.0–13.5] (n = 70)
India	13	112^**^	0.47 [0.22–0.98]	0.55 [0.27–1.75]	0.42 [0.02–1.59]	0.03 [0.02–0.06]	13.9 [13.2–14.5] (n = 67)

*R* (median), median reproduction number across outbreaks, exp(*μ*_*R*_); *R* (mean), mean reproduction number across outbreaks, exp(*μ*_*R*_ + *σ*_*R*_^2^/2); *σ*_*R*_, standard deviation of log(*R*_*j*_) across outbreaks, quantifying between-outbreak heterogeneity; *k*, dispersion parameter, with smaller values indicating greater case-level transmission heterogeneity. CrI, credible interval (2.5th–97.5th posterior percentile); CI, 95% bootstrap confidence interval (1,000 pair-level resamples). All estimates from the hierarchical negative binomial model under prior set P2. * Case count for Faridpur 2010 increased by 1 (16 from Sazzad *et al*. (2013); 17 in the dataset). The article suggested the possibility of a common infector for the two confirmed cases (one admitted patient with no history of date palm sap consumption and one physician). An unidentified NiV-infected case-patient in the same adult male ward, given the context, is considered highly plausible and included in the dataset as an unknown primary case. ** Case count for Siliguri 2001 increased by 1 (66 from Harit *et al*. (2006); 67 in the dataset). The primary case, though unidentified, has a precisely attributed offspring count of 9 from the transmission diagram and text description, but was not counted as a case in the source study. Excluding this case would discard a valid observation of an unusually high offspring count, biasing the offspring distribution toward underdispersion. We therefore include this case as an infector with an offspring count of 9, with an unknown onset date, and an unknown transmission setting (coded accordingly).

#### Country-specific analyses.

Fitting each country separately, the median *R* was 0.47 (0.22–0.98) in India and 0.35 (0.19–0.63) in Bangladesh, and the dispersion parameter *k* was 0.03 (0.02–0.06) and 0.05 (0.03–0.09), respectively (Table 2; Fig 4). Both countries showed high overdispersion (*k* << 1). The posterior probability that *R* was higher in India than Bangladesh was 0.73 (median ratio 1.33 [0.51–3.42]), and the probability of lower *k* in India was 0.85 (median ratio 0.65 [0.26–1.48]). Neither contrast was conclusive. *σ*_*R*_ was prior-sensitive in both countries. India’s median *R* was sensitive to prior specification, ranging from 0.47 (0.22–0.98) under P2 to 0.84 (0.30–3.04) under P1, whereas P2 and P3 gave concordant estimates (0.47 and 0.48), suggesting the India data are insufficient to constrain *R* without informative prior structure. Bangladesh’s *R* was more stable across priors (0.35–0.42). Both countries’ *k* estimates were robust across all prior specifications. More details on country-specific estimates and posterior predictive checks are provided in the Appendix (Text F; Table I; Figs B and F in S1 Appendix).

**Fig 4:**
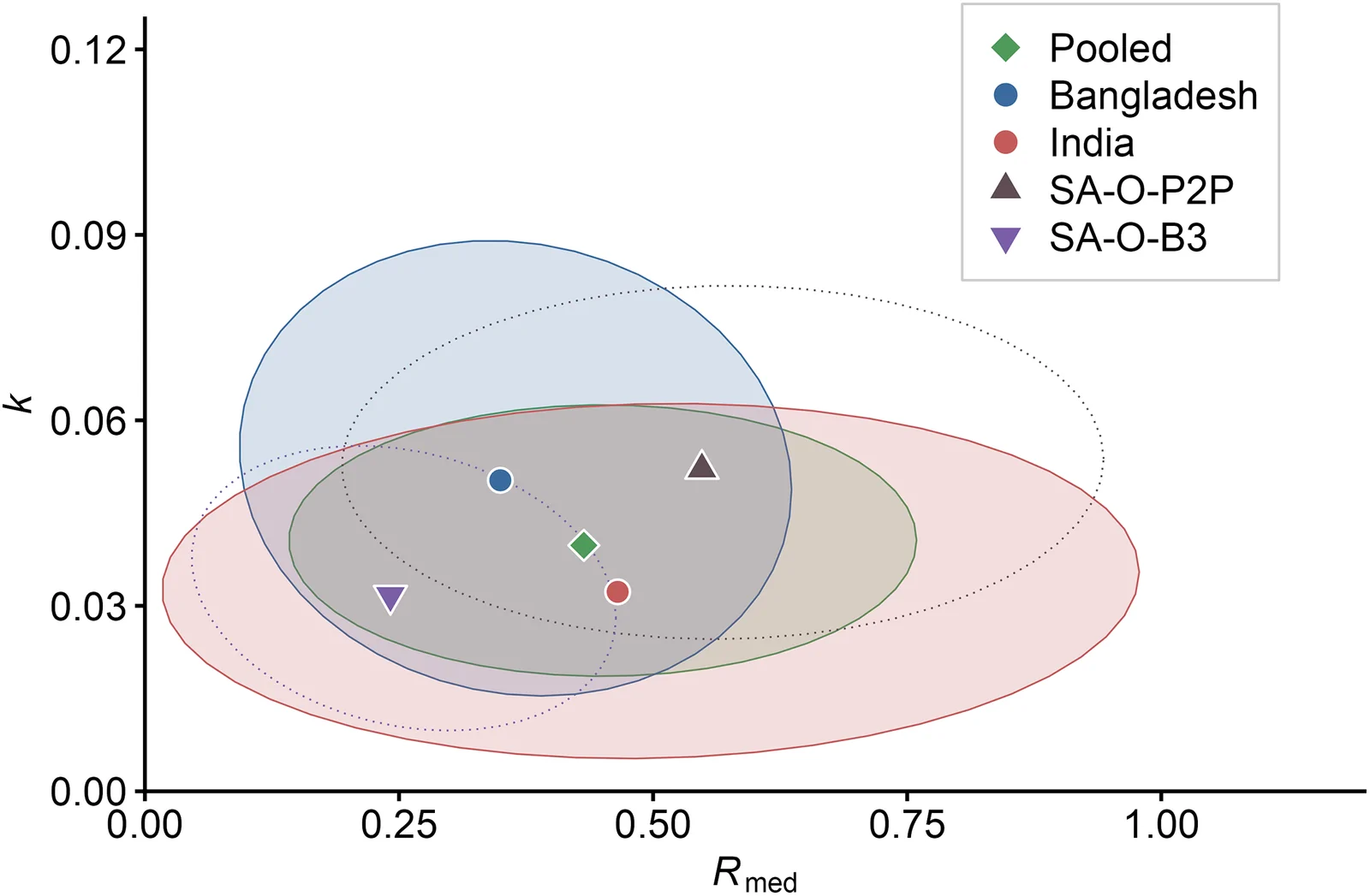
Reproduction number and offspring dispersion. Posterior medians of the reproduction number (*R*_*med*_) and the offspring dispersion parameter (*k*) from independent hierarchical negative binomial fits under prior set P2 (epidemiologically informed, permissive between-outbreak heterogeneity). Diamond: pooled analysis (67 outbreaks, 323 cases). Circles: country-specific analyses (Bangladesh, 54 outbreaks, 211 cases; India, 13 outbreaks, 112 cases). Triangle: sensitivity analysis restricted to person-to-person outbreaks (21 outbreaks, 245 cases; SA-O-P2P). Inverted triangle: sensitivity analysis with augmented Bangladesh dataset (Bangladesh, 191 outbreaks, 348 cases; SA-O-B3), a conservative lower bound on *R*_*med*_. Ellipses denote 95% bivariate normal credible regions of the joint posterior.

#### Sensitivity analyses.

Across all structural and parameterisation sensitivity analyses, *R* (median) remained below 1 and *k* below 0.2 (Fig 4, Table 3). Restriction to outbreaks with person-to-person transmission (SA-O-P2P; 21 outbreaks; 245 cases) yielded *R* (median) = 0.55 (0.33–0.93) and *k* = 0.05 (0.03–0.08). This estimate characterises person-to-person transmissibility, providing an upper bound for transmission potential while remaining subcritical. Excluding the two manually inferred index cases from 2001 Siliguri and 2010 Faridpur outbreaks (67 outbreaks, 321 cases; SA-O-MI) yielded *R* (median) = 0.41 (0.24–0.71) and *k* = 0.04 (0.02–0.06), closely mirroring primary model estimates (*R* (median) = 0.43 [0.26–0.75]; *k* = 0.04 [0.03–0.06]) and suggesting that transmission tree reconstruction choices do not drive the overdispersion estimate. Stratification of the Bangladesh dataset by surveillance era yielded a median *R* of 0.39 (0.17–0.87) in the pre-surveillance period (SA-O-B1; 5 outbreaks, 85 cases) and 0.28 (0.15–0.57) under prospective surveillance from 2007 onward (SA-O-B2; 49 outbreaks, 126 cases). Augmenting the Bangladesh analysis with 137 additional national surveillance cases (IEDCR) [31], assumed to be non-transmitting spillovers (SA-O-B3; 348 cases covering from 2001 to 2026) established a conservative lower bound of *R* (median) = 0.24 (0.13–0.45) and *k* = 0.03 (0.02–0.05), compared with *R* (median) = 0.35 (0.19–0.63) and *k* = 0.05 (0.03–0.09) in the Bangladesh country analysis (Table 2). Reparameterising the offspring variance so that the variance-to-mean ratio was held constant across outbreaks (SA-O-CV) yielded *R* (median) = 0.40 (0.25–0.66) and an implied *k* at the median *R* of 0.04 (0.02–0.06). The fitted *ψ* = 10.22 (5.90–19.08) closely matched the ratio implied by the primary model posterior (*ψ* derived = 10.87 [5.72–22.00]), consistent with the conclusion that subcritical transmission and overdispersion were robust to the dispersion specification (Tables J and K; Text F in S1 Appendix).

**Table 3:** Offspring distribution sensitivity analyses.

Analysis	Outbreaks	Cases	Reproduction number *R* (median) [95% CrI]	Reproduction number *R* (mean) [95% CrI]	Between-outbreak heterogeneity *σ*_*R*_ [95% CrI]	Offspring dispersion parameter *k* [95% CrI]
SA-O-P2P	21	245	0.55 [0.33–0.93]	0.58 [0.36–1.08]	0.24 [0.01–0.89]	0.05 [0.03–0.08]
SA-O-MI	67	321	0.41 [0.24–0.71]	0.45 [0.27–0.88]	0.29 [0.01–1.06]	0.04 [0.02–0.06]
SA-O-B1	5	85	0.39 [0.17–0.87]	0.52 [0.23–2.86]	0.60 [0.02–2.00]	0.04 [0.02–0.09]
SA-O-B2	49	126	0.28 [0.15–0.57]	0.32 [0.17–0.81]	0.36 [0.02–1.24]	0.07 [0.03–0.15]
SA-O-B3	191	348	0.24 [0.13–0.45]	0.28 [0.15–0.64]	0.44 [0.02–1.28]	0.03 [0.02–0.05]

SA-O-P2P: Restriction to outbreaks with person-to-person transmission. SA-O-MI: Excluding two manually inferred index cases (2001 Siliguri and 2010 Faridpur outbreaks). SA-O-B1: Pre-surveillance era (Bangladesh, pre-2007; n = 5 outbreaks); The wide upper bound on *R* (mean) reflects weak identification of *σ*_*R*_ with few units; *R* (median) is the preferred summary for this subset. SA-O-B2: Prospective surveillance era (Bangladesh, 2007+). SA-O-B3: Augmented Bangladesh dataset. *R* (median), median reproduction number across outbreaks, exp(*μ*_*R*_); *R* (mean), mean reproduction number across outbreaks, exp(*μ*_*R*_ + *σ*_*R*_^2^/2); *σ*_*R*_, standard deviation of log(*R*_*j*_) across outbreaks, quantifying between-outbreak heterogeneity; *k*, dispersion parameter, with smaller values indicating greater case-level transmission heterogeneity. CrI, credible interval (2.5th–97.5th posterior percentile).

### Serial interval

The gamma distribution provided the best fit to 137 transmission pairs (ΔAIC versus lognormal = 10.0; versus Weibull = 5.1), yielding a fitted mean serial interval of 13.3 days (95% confidence interval: 12.8–13.8) and standard deviation (SD) of 3.3 days (2.7–3.9). The median of the observed pairs was 13.0 days, close to the fitted mean (Table 2). A kernel density estimate of the observed distribution is shown alongside the fitted gamma curve in Fig 5. Country-specific estimates were consistent with the pooled results: 12.8 days (12.0–13.5) in Bangladesh (n = 70 pairs) and 13.9 days (13.2–14.5) in India (n = 67 pairs). Bangladesh-specific estimate was consistent with the previously reported mean of 12.7 days (SD: 3.0) by Nikolay *et al*. [6]. Sensitivity analyses yielded consistent estimates across all specifications (SA-S1 and SA-S2), with mean serial intervals ranging from 12.6 to 13.4 days regardless of pair inclusion criteria or surveillance era (Table L in S1 Appendix).

**Fig 5:**
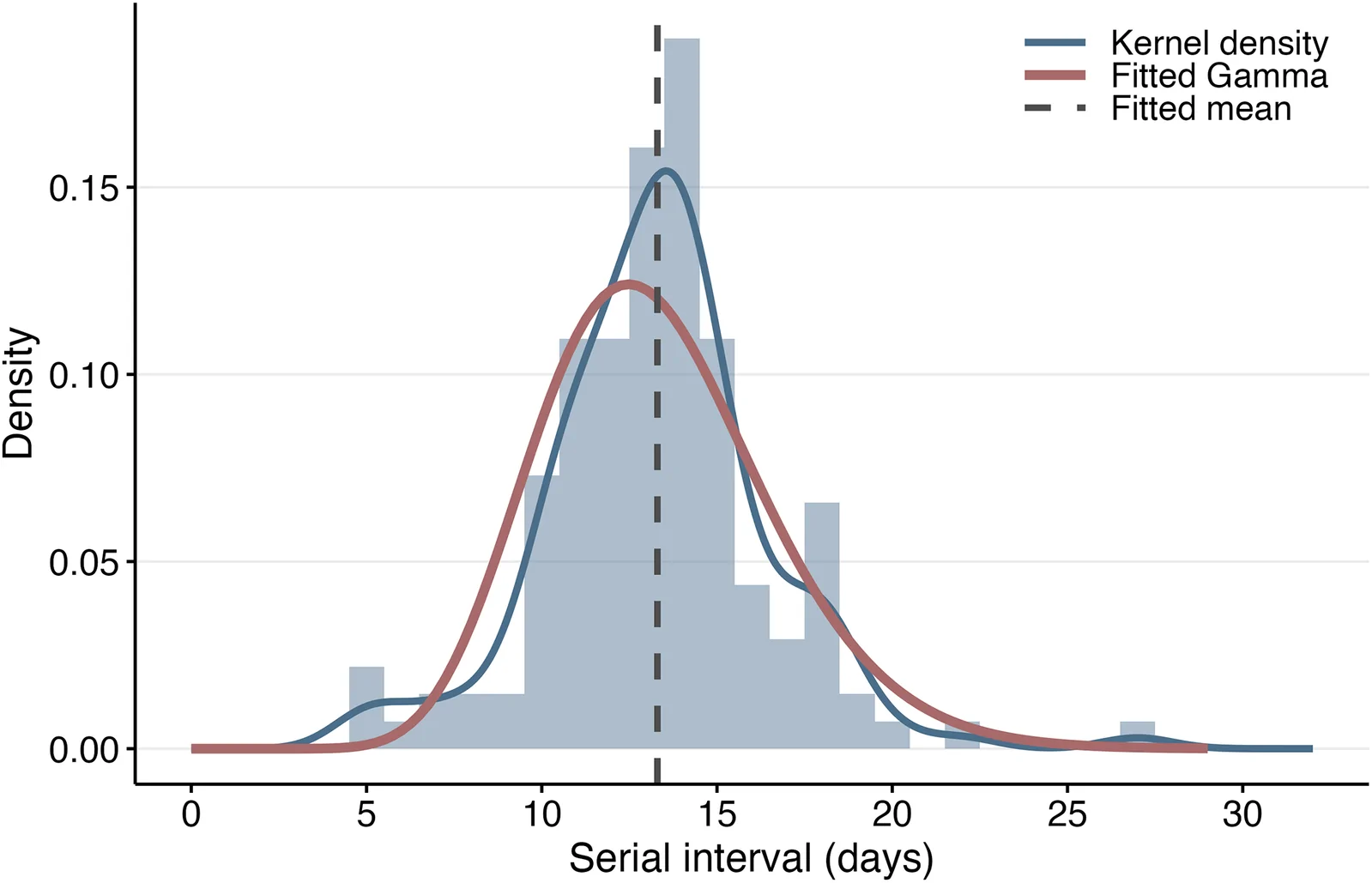
Serial intervalinterval. Distribution of observed serial intervals (n = 137 transmission pairs) with fitted gamma density curve (red) and kernel density estimate (KDE; dark blue line). The dashed vertical line indicates the fitted mean (13.3 days). The gamma distribution provided the best fit among three candidate distributions (ΔAIC versus lognormal = 10.0; versus Weibull = 5.1), with a fitted mean of 13.3 days (95% CI: 12.8–13.8) and standard deviation of 3.3 days. The KDE shows the empirical distribution is slightly more concentrated around the mean than the fitted gamma curve; the fitted and empirical standard deviations are closely matched (3.3 versus 3.2 days), suggesting the overall spread is well captured.

## Discussion

We reviewed and analysed outbreak data from Nipah virus outbreaks in Bangladesh and India spanning 2001–2026 and estimated the reproduction number, offspring dispersion, and serial interval that characterise NiV transmission. The findings suggest that the transmission is subcritical on average, with a reproduction number well below the epidemic threshold (*R* = 1); individual-level transmission is markedly overdispersed, so a minority of cases drives most onward transmission; and the mean serial interval is 13.3 days. Together, these describe transmission that is self-limiting on average but capable of stochastic amplification, in which a low reproduction number should not be equated with low risk.

The subcritical reproduction number implies that NiV transmission following zoonotic introduction can be expected to die out under current levels of transmissibility and virulence, and that sustained transmission is unlikely without repeated reintroduction. The low average reproduction number, however, should be understood alongside the dispersion parameter: most introductions die out quickly, but a minority can generate large clusters. This reconciles the two key features of NiV outbreaks, frequent dead-end spillovers and occasional larger clusters. While these transmission dynamics are consistent with prior evidence from Bangladesh [6,7,24,36], our analyses provide a formal quantitative evaluation encompassing both Bangladesh and India over a twenty-five-year period since NiV emergence in 2001 until 2026.

Bangladesh has operated a continuous, national NiV-specific hospital-based surveillance system since 2007 [6,7,63]. An independent evaluation of this system estimated that approximately half of outbreaks were missed during 2007–2014, with no association found between outbreak size and distance from a surveillance hospital, the primary driver of detection in their model [63], arguing against underdetection of isolated spillovers specifically. India lacked a comparable system for most of the study period, with cases instead identified reactively following outbreaks [64], and surveillance capacity has varied within India across settings and over time [65,66]. Because a substantial share of India’s person-to-person outbreaks were detected reactively, only cases severe or outbreaks large enough to be recognised may be disproportionately represented, which could inflate the degree of overdispersion in India. The available data, however, cannot distinguish a genuine country-level difference in transmission from this ascertainment effect.

Our estimated median reproduction number in Bangladesh closely matches the previous estimate published by Nikolay *et al*. [6]. Our sensitivity analysis with augmented data for Bangladesh is similarly consistent with a recent estimate derived from surveillance data covering 2007–2018 [24], supporting the validity of both our model structure and dataset. Overdispersion in Bangladesh had previously been documented descriptively [6,24,36], and the dispersion parameter for Bangladesh has been used by Nikolay *et al*. 2021 and Cortes-Azuero *et al*. 2026 [67,68]. Pan *et al*. conducted an analysis using a next-generation cluster approach fitted to a smaller dataset across Bangladesh and India [69], overlapping our estimate. Further, we generated comprehensively synthesised country-specific estimates of reproduction number, offspring distribution, and serial interval for India.

Our estimates carry implications for NiV prevention and control. Because transmission is self-limiting and concentrated in a minority of cases, targeted non-pharmaceutical containment such as rapid contact tracing and quarantine is expected to be more efficient than population-wide measures [70,71]. Further, the reproduction number, offspring dispersion parameter, and serial interval estimated across Bangladesh and India provide empirical inputs for broader epidemiological modelling. These parameters can be leveraged to forecast outbreak trajectories, estimate the probability of nosocomial clusters, and evaluate the effectiveness of non-pharmaceutical interventions across country settings.

The reproduction number, offspring dispersion parameter, and serial interval provide key transmission parameters to assess operational constraints for deploying and evaluating medical countermeasures. While a 13.3-day serial interval, which serves as a proxy for the generation interval, outlines a temporal window for ring-based control; recent modelling studies indicate that reactive ring vaccination yields limited impact or feasibility under current NiV epidemiology [67,68,72]. Additionally, as demonstrated by Hinch *et al*. (2026), pronounced overdispersion severely reduces the statistical power of standard cluster-randomised ring trials [73]. Thus, establishing vaccine efficacy under current NiV epidemiology will likely necessitate alternative trial designs that accommodate the degree of overdispersion of NiV, or resort to regulatory pathways that do not depend on clinical disease endpoints, such as licensure based on animal model efficacy data.

Our estimates suggest that, because human transmission chains are expected to extinguish, the burden of NiV disease is driven primarily by the frequency of zoonotic spillover. Preparedness should therefore combine control of human transmission with interventions that reduce spillover at the animal-human interface, including safer date palm sap harvesting and One Health surveillance of bat populations. Because the risk is regional rather than nationally bounded, as illustrated by the concurrent appearance of cases in West Bengal and Bangladesh in January 2026 [57,62], preparedness also calls for harmonised cross-border surveillance, shared case definitions, and coordinated genomic and epidemiological data collection across the Nipah belt spanning the two countries. The estimates reported here also provide a baseline against which future shifts in transmissibility, such as through viral evolution, can be identified.

Our study has limitations. Reliance on published investigations introduces ascertainment and reporting biases, most notably the absence of outbreak-level Bangladesh data for 2015–2022. The impact of the missing cases was explored in a sensitivity analysis (SA-O-B3) that included cases with no associated outbreak record, assumed to be non-transmitting spillover introductions. Mild or asymptomatic infections might not be captured in outbreak investigations. The estimates also rest on the assumptions of the hierarchical negative binomial framework, which specifies a lognormal mixing distribution for outbreak-specific reproduction numbers and conditional independence of offspring counts given the outbreak-level parameters. The mixing distribution was not varied in the sensitivity analyses, though the small number of outbreaks and the prior-dominated between-outbreak variance make the estimates unlikely to be sensitive to this choice. Conditional independence may be approximate within outbreaks, where offspring counts are linked through the shared transmission process. These limitations are more likely to affect the precision of individual estimates and the country comparison than the central conclusions, which were consistent across prior specifications and sensitivity analyses, including exclusion of the manually inferred cases and the augmented Bangladesh analysis.

In summary, NiV in Bangladesh and India operates in a subcritical, highly overdispersed transmission regime in which individual outbreaks remain self-limiting, yet the capacity for stochastic superspreading, combined with high case-fatality and recurrent spillover, generates persistent public health risk. Further, our estimates of reproduction number, offspring dispersion, and serial interval provide the quantitative foundation for epidemiological modelling of NiV outbreaks and for assessing the potential impact of NiV vaccines in the development pipeline.

## Supporting information

S1 Appendix Text A. Choosing studies describing the same outbreaks. Text B. Analytical dataset. Text C. Coding decisions. Text D. Offspring distribution – prior specifications and sensitivity. Text E. Offspring distribution – sensitivity analyses. Text F. Results. Text G. Reproduction number and overdispersion parameter – existing estimates from literature. Table A. PRISMA Checklist. Table B. Search strategies for each database. Table C. Inclusion and exclusion criteria. Table D. List of data extracted. Table E. Studies included in the systematic review. Table F. Quality assessment results. Table G. Person-to-person outbreaks by transmission chain. Table H. Prior sensitivity. Table I. Country-specific analyses: P1–P3. Table J. Offspring distribution sensitivity analyses (SA-O-CV). Table K. Derived dispersion quantities from primary model. Table L. Serial interval: primary and sensitivity analyses. Fig A. Epidemic curves of Nipah virus outbreaks in Bangladesh and India. Fig B. Prior and posterior densities under three prior specifications. Fig C. Posterior predictive check. Fig D. Observed and simulated offspring count distributions. Fig E. Trace plots. Fig F. Posterior predictive check by country.
(DOCX)
